# Bioindicator potential of *Ricinus communis* to simulated rainfall containing potassium fluoride

**DOI:** 10.7717/peerj.9445

**Published:** 2020-07-01

**Authors:** Douglas Almeida Rodrigues, Juliana de Fátima Sales, Sebastião Carvalho Vasconcelos Filho, Arthur Almeida Rodrigues, Eduardo Matheus Guimarães Teles, Alan Carlos Costa, Efraim Lázaro Reis, Thais Andrade de Carvalho Silva, Caroline Müller

**Affiliations:** 1 Laboratory of Seeds, Goiano Federal Institute of Education, Science and Technology, Rio Verde, Goiás, Brazil; 2 Laboratory of Plant Anatomy, Goiano Federal Institute of Education, Science and Technology, Rio Verde, Goiás, Brazil; 3 Laboratory of Ecophysiology and Plant Productivity, Goiano Federal Institute of Education, Science and Technology, Rio Verde, Goiás, Brazil; 4 Department of Chemistry, Federal University of Viçosa, Viçosa, Minas Gerais, Brazil

**Keywords:** Fluoride, Pollution, Tolerant plants, Castor plant

## Abstract

**Background:**

Fluoride pollution is a global problem because of its high phytotoxicity. Fluoride is released in air, water and soil through industrial processes, where it damages various plant species. *Ricinus communis* is widely distributed in Brazil, India and China and has been extensively used as a phytoremediation species in heavy metal-contaminated soils. However, few studies regarding the effect of air pollutants on *R. communis* have been published, and no information about the exposure of this species to fluoride is available. Therefore, the aim of the present study was to investigate the effects of fluoride on *R. communis* morphoanatomical and physiological responses using simulated rainfall containing potassium fluoride (KF).

**Methods:**

Young plants at approximately 10 days after emergence were treated daily with KF using simulated rainfall at 0, 1.5, 3.0 and 4.5 mg L^−1^, for 37 consecutive days. Chlorophyll *a* fluorescence, gas exchange, anatomical characteristics and fluoride accumulation in the roots and leaves were evaluated after this period.

**Results:**

No visual or anatomical symptoms were observed for the first three treatments. Necrosis and chlorosis were visually evident after the 37th day of KF application at 4.5 mg L^−1^, followed by changes in parenchyma tissues, cell collapse and phenolic compound accumulation at the end of the experiment. No damage was observed in terms of photosynthetic photochemical and biochemical stages. Maintenance of physiological characteristics in the presence of fluoride accumulation in roots and leaves were shown to be important fluoride biomarkers. These characteristics suggest that *R. communis* is tolerant to 1.5 and 3.0 mg L^−1^ KF, and is anatomically sensitive at 4.5 mg L^−1^ KF.

## Introduction

The incidence of environmental air pollution has increased simultaneously to industrial progress (Li, Li & Zhang, 2018). Fluoride (F) is one of the most phytotoxic contaminants (Panda, 2015). High F concentrations are released into the environment as a result of several anthropogenic activities, including aluminum smelting (Choubisa & Choubisa, 2016), coal burning (Ding et al., 2013) brick manufacturing (Jha et al., 2008), direct application of phosphate fertilizers (Ramteke et al., 2018) and fluoridated water irrigation, which is also a source of diffuse soil fluoride (Fawell et al., 2006). In the atmosphere, F may be released in both gaseous and liquid forms at concentrations ranging from 0.01 to 10 mg L^−1^ (Smith & Hodge, 1979). F concentrations in agricultural soils range from 100 to 5,300 mg kg^−1^ (Mikkonen et al., 2018; Singh et al., 2018). The World Health Organization (WHO) has set a limit for fluoride in drinking water of 1.5 mg L^−1^. Nevertheless, F levels in aquifers and water bodies often exceed this value (Silva, 1983; Martins et al., 2018). In various parts of the world, fluoride concentrations in water from aquifers used for plant irrigation range from 1.5 to 5.0 mg L^−1^ (Vikas et al., 2013; Abiye, Bybee & Leshomo, 2018).

Because of its high toxicity, F affects biodiversity. In the case of plants, F is absorbed by root tissues and is retained both in the cell wall and in the intracellular space, restricting translocation to aerial parts, mainly in tolerant species (Zouari et al., 2017). When F is present in its gaseous form in the atmosphere, it is absorbed through the stomata and cuticles (Sant’Anna-Santos et al., 2014); in aqueous solutions, it can be incorporated by the entire entrance path to the leaf surface (Anjos et al., 2018). Upon penetrating leaves, F moves through apoplastic pathways, reaching leaf margins and apices, and may also accumulate in the mesophyll, leading to lesions such as parenchyma cell collapse, resulting in chlorosis and necrosis (Sharma & Kaur, 2018) as well as changes in primary plant metabolism, including the photosynthetic process (Rodrigues et al., 2018a).

In order to determine the effects of F on plant development, it is necessary to recognize the relationship between F and particular species, and estimate anatomical and physiological visual damage to leaves and roots to determine sensitivity or tolerance. Sensitive plants are used to directly determine biological effects caused by pollutants as well as early pollutant damage through laboratory assays, which are relatively low-cost compared to technical measurement methods (Oguntimehin, Kondo & Sakugawa, 2010).

Most crops require agricultural irrigation with groundwater or are exposed to direct contact with air pollutants. *Ricinus communis*, popularly known as castor bean, is a cultivar belonging to the Euphorbiaceae family (Salihu, Gana & Apuyor, 2014). It displays high tolerance to diverse environmental conditions, is easily cultivated in tropical climates, and presents low resource requirements, that is, fertile soil. As a result, it is widely distributed, being found in countries such as Brazil, India, Italy and China (Atabani et al., 2013). *R. communis* displays high economic potential because of its oil content and biodiesel production potential (Saez-Bastante et al., 2015). It also serves as a source of raw material for paints, cosmetics, varnishes, lubricants and drugs (Barbosa et al., 2010). Furthermore, *R. communis* is widely used for the phytoremediation of heavy metals, including Cd, Zn and Cu (Wang et al., 2016), and has been reported as tolerant to atmospheric pollutants, including SO_2_ (Singh et al., 1991), although it is sensitive to O_3_ (Rathore & Chaudhary, 2019). To the best of our knowledge, no studies on the effects of F on *R. communis* morphology, anatomy and physiology traits have been reported until now. Therefore, this study assessed the effects of F on morphological and physiological responses in *R. communis* using simulated rainfall containing potassium fluoride (KF). The potential of this species as a bioindicator for KF was also evaluated.

## Materials and Methods

### Plant material, growing conditions and KF treatments

*R. communis* seeds were obtained from 50 adult plants in full production through manual harvesting with pruning shears and manual depulping. The EVF 712 plant genotype from Israel was used. Initially, seeds were treated with Vitavax^®^-Thiram fungicide (30%) and were later seeded in five L containers with Bioplant^®^ substrate containing the following nutrient concentrations: F—25 mg kg^−1^; N—8.6 g kg^−1^; P—0.2 g kg^−1^; K—0.3 g kg^−1^; Ca—1.3 g kg^−1^; Mg— 2.9 g kg^−1^; and S—0.9 g kg^−1^. Four seeds per pot were sown. The experiment was performed under controlled conditions in a greenhouse located at the Goiano IF, Brazil (latitude 17° 48′ 16″ S, longitude 50° 54′ 19″ W and altitude of 753 m). Environmental parameters were monitored using a model-32 SKDL data logger with a temperature and relative humidity sensor. The average relative humidity was 65% (± 5) and average temperatures were 29° C (± 5 day) and 25 °C (± 5 night).

Approximately 10 days after emergence, plants with a standardized height (~15 cm) with at least four leaves were chosen, leaving two plants per pot. Subsequently, the plants were exposed to a liquid KF solution (pH 6.0) at 0 (control), 1.5; 3.0 and 4.5 mg L^−1^ to simulate constant fluoride release in the vicinity of polluted areas (Smith & Hodge, 1979). The pH values of the solutions were adjusted using HCl (2.0 M) and NaOH (2.0 M). F application was also performed simulating rainfall with manual sprays, applying 250 mL day^−1^ per pot, sufficient to wet the entire plant surface. KF was applied from top to bottom, dripping on leaves and then flowing to the substrate, simulating exposure to the pollutant in natural conditions. After 37 days of KF exposure, visual, physiological and anatomical assessments were performed.

A completely randomized design was carried out consisting of four treatments (KF concentrations) and four replicates, each replicate composed of two plants.

### Foliar symptoms

Visual symptoms were recorded by photographing the leaf surface of fully expanded *R. communis* leaves at the end of the experimental period, using a digital camera (Cyber-Shot SONY HX100V, Japan). The leaf with the greatest homogeneity compared to the remaining leaves of each treatment was selected for photographing.

### Morphoanatomical root and leaf characterizations

For the morphoanatomical analyses, median root regions (1 cm) and leaf cross sections (0.5 cm^2^) from the median region of the third or fourth fully-expanded leaf from all the replicates were collected. The plant material was prepared for historesin infiltration as detailed by Rodrigues et al. (2018b). The samples were sectioned at 5-μm thickness on a rotary microtome and each section was stained with toluidine according to O’Brien, Feder & Mccully (1964). Photographs were taken using a DP-72 camera coupled to an Olympus microscope (BX61, Tokyo, Japan). Micromorphometric measurements of the adaxial and abaxial face epidermises of the mesophyll, spongy and palisade parenchyma were performed using ImageJ software (Image Processing and Analysis in Java, v. 1.47, USA) on ten observations per repetition.

Starch location was also identified by histochemical staining using Lugol solution at 10 g L^−1^ (Jensen, 1962). The calculations of the percentage areas marked by Lugol were performed by assessing contrast difference using the ImageJ software.

### Gas exchanges

The net photosynthetic rate (*A*, μmol CO_2_ m^−2^ s^−1^), transpiration rate (*E*, mmol H_2_O m^−2^ s^−1^) and internal CO_2_ concentration (Ci, μmol mol^−1^) were measured using an infrared gas analyzer (IRGA, model LI-6400XTR, LI-COR, Lincoln, NE, USA) in fully expanded leaves under active photosynthetically radiation (PAR) (1,500 μmol photons m^−2^ s^−1^) and CO_2_ concentration (400 μmol mol^−1^) constants, and environment temperature (~27 °C) and relative humidity (~52%). The photosynthetic rate/internal concentration of carbon dioxide ratio (*A/C_i_*) and the electron transport rate/carbon dioxide assimilation (ETR/*A*) were calculated according to Ribeiro et al. (2009). Respiratory rates (*R*_*D*_, μmol CO_2_ m^−2^ s^−1^) were measured in dark conditions to calculate the maximum quantum yield of CO_2_ assimilation (Φ_CO2_) according to Fryer et al. (1998).

### Chlorophyll *a* fluorescence

Chlorophyll *a* fluorescence was evaluated using a 6400-40 LCF fluorometer coupled to the IRGA, to obtain the minimum (*F*_0_) and maximum (*F*_m_) fluorescence, the potential (*F*_v_/*F*_m_) and effective (Φ_PSII_) quantum yield of the PSII, the apparent electron transport rate (ETR), photochemical quenching (qP), and the non-photochemical quenching (qN), as detailed by Rodrigues et al. (2019).

### Fluoride content

Fluoride content was measured in leaf and root samples after 24 h of the last simulated KF rainfall. The samples were dried, ground and the fluoride extracted according to Frant & Ross (1968), with modifications (Anjos et al., 2018). Fluoride content was measured using a potentiometer (model 8519; Hanna Instruments®) and expressed as µg g^−1^.

### Statistical analyses

The confirmed data for normality of errors (Shapiro–Wilk) and homogeneity of variances (Levene) were subjected to analysis of variance (ANOVA), followed by comparison of means by Dunnett’s test, considering the significance levels of 1% (**) and 5% (*). All statistical analyses were performed using ASSISTAT v. 7.7 software.

## Results

### Morphological traits

**Figure 1:**
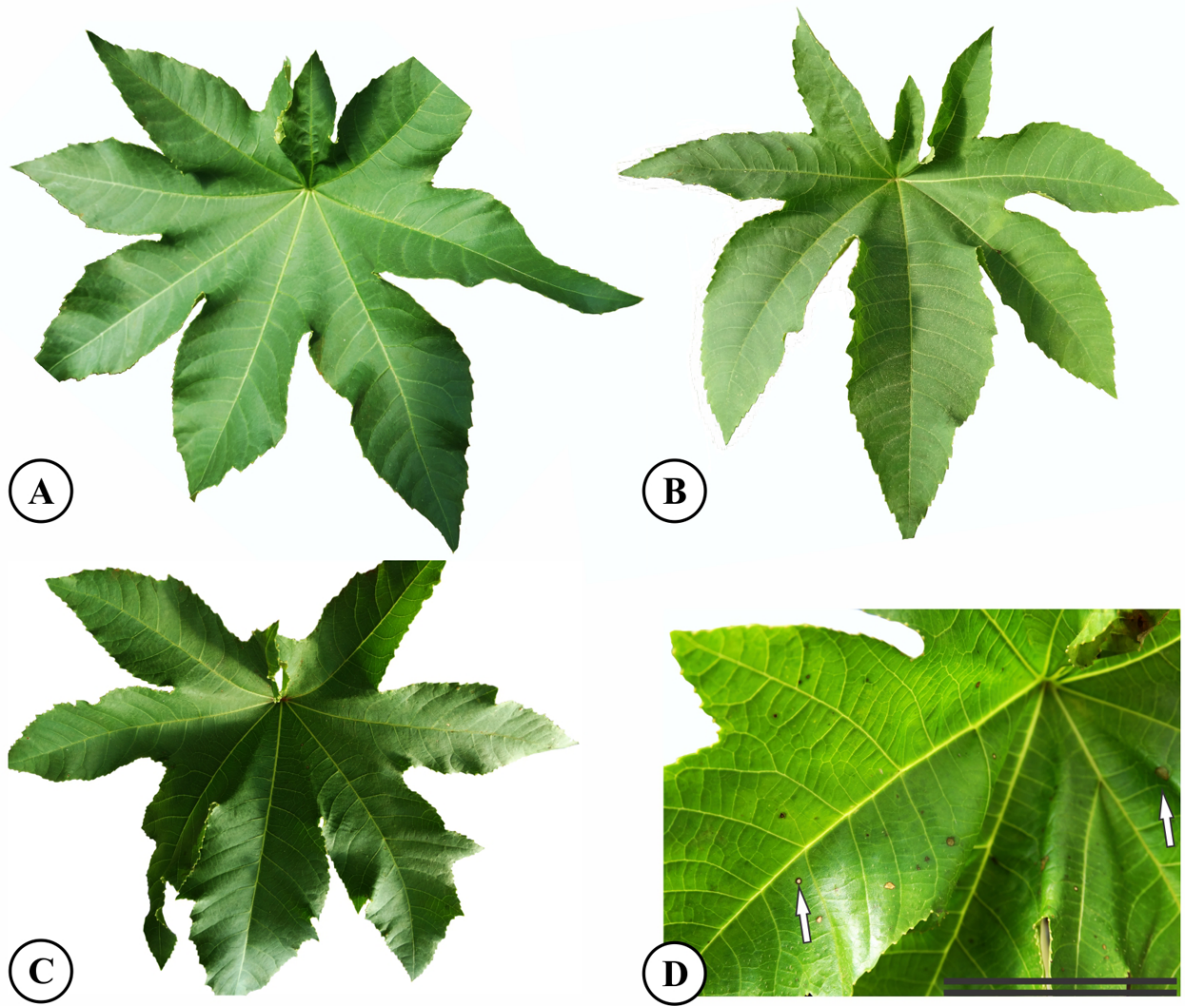
Symptoms of fluoride toxicity in *Ricinus communis* leaves highlighted by white arrows after 37 days of exposure. (A) Control, (B) 1.5 mg L^−1^ KF, (C) 3.0 mg L^−1^ KF (D) 4.5 mg L^−1^ KF. Scale bar: 5 cm.

After 37 days of potassium fluoride application, no differences in symptoms were observed between the control treatments and 1.5 or 3.0 mg L^−1^ KF (Figs. 1A–1C). However, plants treated with 4.5 mg L^−1^ KF displayed chlorotic pigment formation, in brown tones, on small parts of the leaf surface (Fig. 1D). No plant deaths were observed in any of the KF treatments.

### Anatomical changes

**Figure 2:**
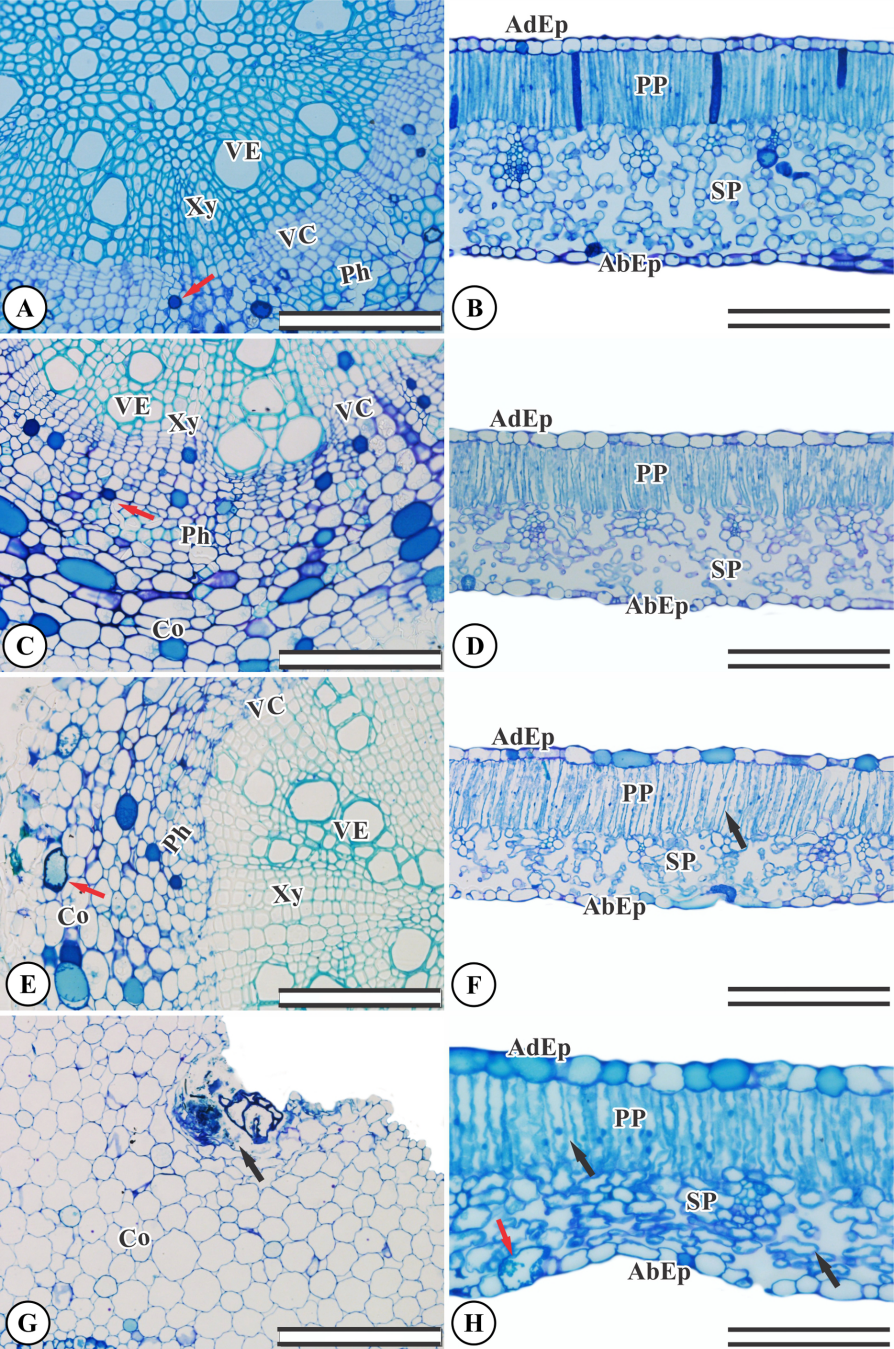
Sections from *Ricinus communis* roots and leaves after 37 days of exposure to fluoride. (A and B) Control, (C and D) 1.5 mg L^−1^ KF, (E and F) 3.0 mg L^−1^ KF and (G and H) 4.5 mg L^−1^ KF. (VE) vessel element. (Xy) xylem. (VC) vascular cambium. (Ph) phloem. (Co) cortex. (AdEp) adaxial epidermis. (AbEp) abaxial epidermis. (PP) palisade parenchyma. (SP) spongy parenchyma. Black arrows indicate cell collapse. Red arrows indicate phenolics accumulation. (A, C, E and G) root. (B, D, F and H) leaf. Scale bar: 200 μm.

*Ricinus communis* presents an organized, undamaged adaxial and abaxial epidermis (Fig. 2B). The chlorophyl parenchyma is heterogeneous of the dorsiventral type. The cells of the palisade parenchyma are organized by an integrated layer of elongated cylindrical cells. The spongy parenchyma consists of five to eight layers of polyhedral cells (Fig. 2B). The root anatomy of *R. communis* presents a secondary xylem with intact vessel elements and fibers and both solitary and multiple radial vessels, with an organized vascular cambium and intact secondary phloem containing some cells with phenolic content (Fig. 2A). KF application by simulated rainfall did not alter root and leaf structures in the control group or the 1.5 mg L^−1^ KF treatment (Figs. 2A–2D). In addition, the highest KF doses (3.0 and 4.5 mg L^−1^) did not affect secondary xylem root cells, and vessel elements and fibers were not altered. On the other hand, the vascular cambium, cortex and mainly, parenchymal cells, became disorganized (Figs. 2E–2G). In leaves, KF treatment altered the palisade and spongy parenchyma, resulting in cell wall deformities and increased intercellular spaces in palisade parenchyma cells (Fig. 2F). In addition, the 4.5 mg L^−1^ KF treatment promoted bulging in the spongy parenchyma and in the epidermis abaxial face of the epidermis, where cells presented a sinuous wall and compacted tissue (Fig. 2H).

*Ricinus communis* plants displayed reduced thickness in the spongy and mesophyll parenchyma tissues when exposed to 3.0 and 4.5 mg L^−1^. At the highest dose, a 32% reduction in palisade parenchyma thickness was observed when compared to the control group (Table 1).

**Table 1:** Morphoanatomical measurements in *Ricinus communis* after 37 days of exposure to simulated rainfall containing KF (0, 1.5, 3.0 and 4.5 mg L^−1^). Adaxial epidermis (EpAd), abaxial epidermis (EpAb), palisade parenchyma (PP), spongy parenchyma (SP) and mesophyll (Me).

KF (mg L^−1^)	EpAd (µm)	EpAb (µm)	PP (µm)	SP (µm)	Me (µm)
0	16.77 ± 0.46	13.56 ± 1.20	90.08 ± 1.84	106.26 ± 7.15	204.94 ± 11.52
1.5	16.17 ± 0.46	13.42 ± 1.30	78.30 ± 8.04	95.59 ± 2.68	182.31 ± 5.25
3.0	17.58 ± 0.44	12.32 ± 0.44	86.79 ± 2.42	81.25**± 3.63	168.13**± 1.71
4.5	18.04 ± 0.57	14.27 ± 0.19	60.84** ± 1.75	68.73**± 3.60	127.18**± 6.50
One-way ANOVA					
*F* (*t*-test)	2.97^NS^	0.77^NS^	8.89**	12.76**	20.82**
*p*	0.0746	0.5331	0.0022	0.0004	<0.0001

**Notes:**

** Asterisks indicate significant differences at 1% of probability, relative to the control by Dunnett’s test.

NS Indicates non-significance.

Mean ± SEM (*n* = 4).

### Starch accumulation

**Figure 3:**
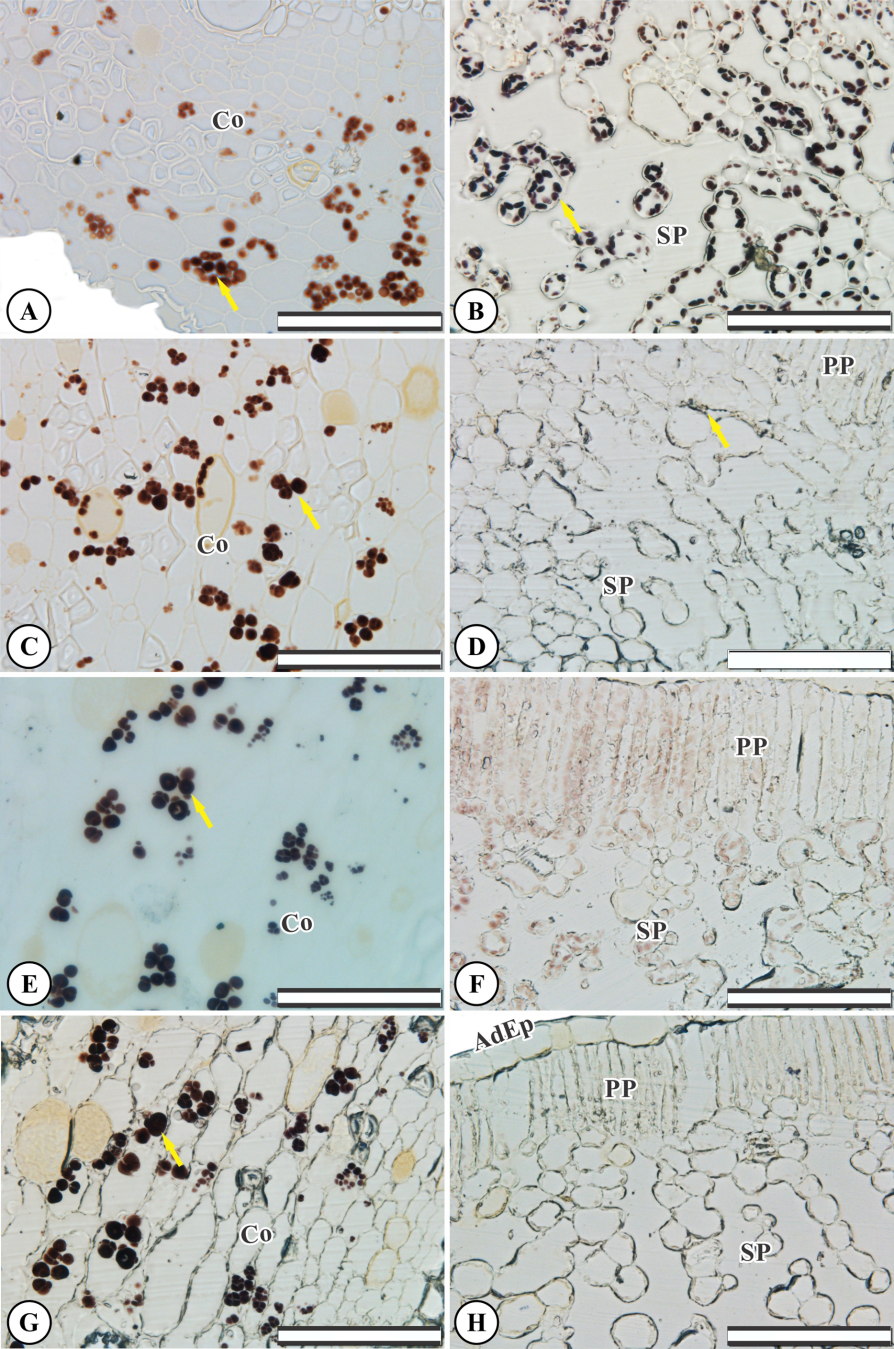
Starch accumulation marked in black in *Ricinus communis* roots and leaves after 37 days of exposure to fluoride. (A and B) Control, (C and D) 1.5 mg L^−1^ KF, (E and F) 3.0 mg L^−1^ KF and (G and H) 4.5 mg L^−1^ KF. (Co) cortex. (AdEp) adaxial epidermis. (PP) palisade parenchyma. (SP) spongy parenchyma. Yellow arrows indicate starch accumulation. (A, C, E and G) root. (B, D, F and H) leaf. Scale bar: 200 μm.

**Figure 4:**
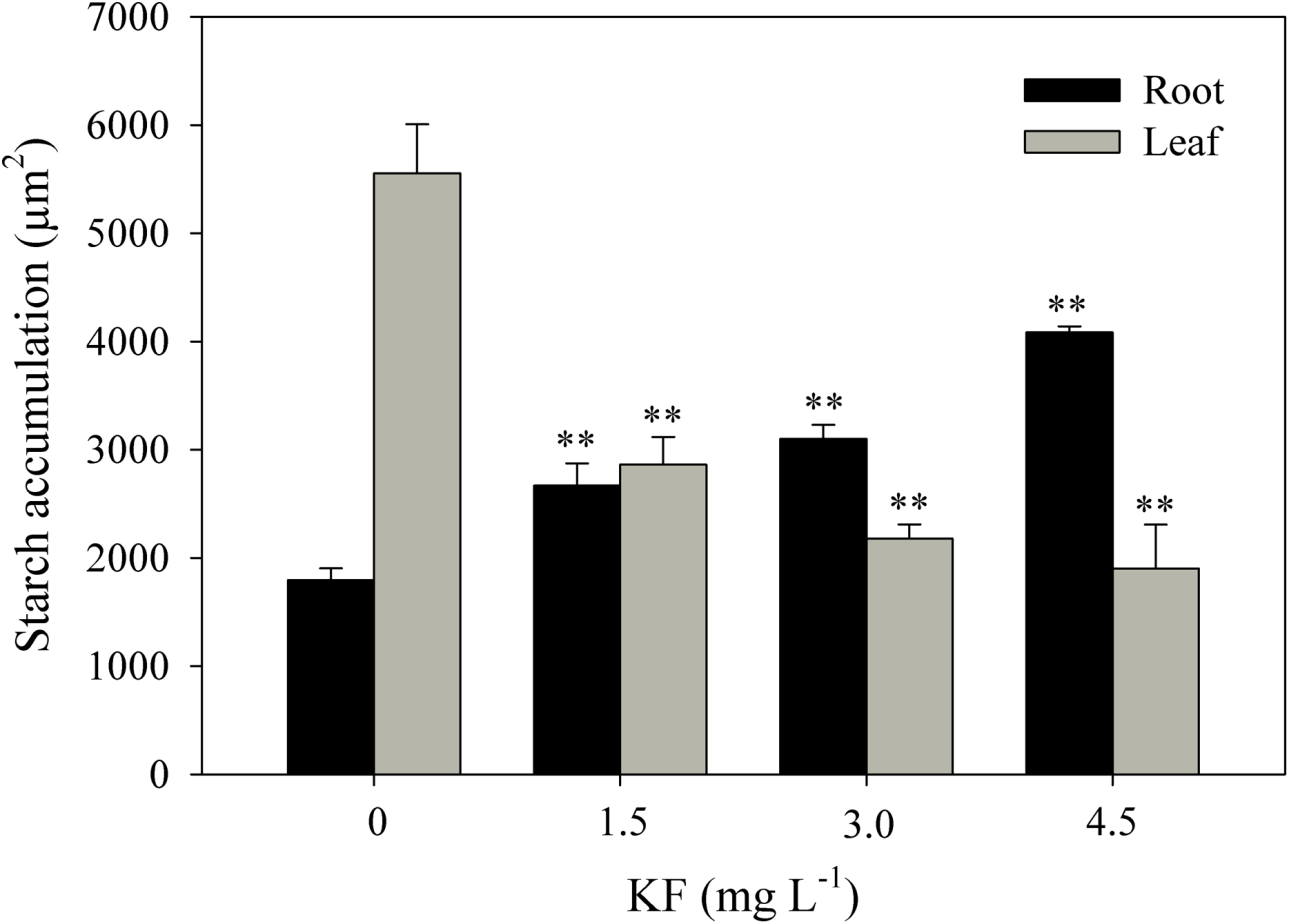
Starch accumulation in *Ricinus communis* roots and leaves after 37 days of exposure to fluoride. Asterisks indicate significant differences at 1% (**) probability, relative to the control by Dunnett’s test. One-way ANOVA Root (*F*-value 48.97**; *p* < 0.0001) and Leaf (*F*-value 24.62**; *p* < 0.0001). Bars represent the mean ± SEM (*n* = 4).

Control root and leaf cells displayed large Lugol-marked areas (Figs. 3A and 3B). In KF-treated plants, the highest starch accumulation was noted in roots, identified by black staining in both epidermal and parenchymatic cells, with increasing KF doses (Figs. 3C, 3E and 3G), resulting in 49, 73 and 128% increments in the marked area when compared to the control (Fig. 4). However, a decrease in starch accumulation with increasing KF doses in leaves was noted (Figs. 3D, 3F and 3H), resulting in 52, 76 and 87% reductions in the marked area (Fig. 4).

### Gas exchanges and chlorophyl *a* fluorescence

Net photosynthetic rate (*A*), transpiration rate (*E*), maximum quantum yield of CO_2_ assimilation (Φ_CO2_) and the ratio between the photosynthetic rate and internal CO_2_ concentrations (*A/*C_i_) and apparent electron transport rate and CO_2_ assimilation (ETR*/A*) were not affected by KF exposure (Table 2).

**Table 2:** Gas exchange measurements in *Ricinus communis* after 37 days of exposure to simulated rainfall containing KF (0, 1.5, 3.0 and 4.5 mg L^−1^). Photosynthetic rate (*A*), transpiration rate (*E*), relation of the photosynthetic rate between the internal CO_2_ concentrations (*A/C_i_*), relation between the apparent electron transport rate and CO_2_ assimilation (*ETR*/*A*) and maximum quantum yield of CO_2_ assimilation (Φ_CO2_).

KF (mg L^−1^)	*A*	*E*	*A/C*_*i*_	ETR/ *A*	Φ_CO2_
0	21.99 ± 0.96	0.005 ± 0.001	0.096 ± 0.005	6.92 ± 0.53	0.018 ± 0.001
1.5	17.41 ± 1.91	0.005 ± 0.001	0.071 ± 0.008	6.88 ± 0.67	0.015 ± 0.002
3.0	19.46 ± 1.23	0.007 ± 0.001	0.070 ± 0.008	5.82 ± 0.55	0.016 ± 0.001
4.5	19.03 ± 1.64	0.006 ± 0.002	0.072 ± 0.009	6.20 ± 0.28	0.016 ± 0.001
One-way ANOVA					
*F* (*t*-test)	1.63^NS^	0.70^NS^	2.62^NS^	1.03^NS^	1.63^NS^
*p*	0.2344	0.5679	0.0988	0.412	0.2342

**Notes:**

NS Non-significant.

Means ± SEM (*n* = 4).

Dunnett’s test.

Concerning chlorophyl *a* fluorescence parameters, only non-photochemical quenching (qN) was significantly altered in *R. communis*, with an increase in 15.39% in the 3.0 mg L^−1^ KF treatment when compared to the control (Table 3). *F*_v_/*F*_m_, Φ_PSII_, ETR and qP did not exhibit any differences in KF treatments compared to the control.

**Table 3:** Chlorophyll *a* fluorescence measurements *Ricinus communis* after 37 days of exposure to simulated rainfall containing KF at different concentrations (0, 1.5, 3.0 and 4.5 mg L^−1^). Potential quantum yield of the PSII (*F*_v_/*F*_m_), effective quantum yield of the PSII (Φ_PSII_), electron transport rate (ETR), photochemical quenching (qP) and non-photochemical quenching (qN).

KF (mg L^−1^)	*F*_*v*_/*F*_*m*_	Φ_PSII_	ETR	qP	qN
0	0.87 ± 0.01	0.24 ± 0.01	150.79 ± 6.11	0.44 ± 0.02	2.21 ± 0.03
1.5	0.87 ± 0.01	0.19 ± 0.01	118.46 ± 12.59	0.36 ± 0.04	2.10 ± 0.05
3.0	0.84 ± 0.02	0.18 ± 0.02	114.45 ± 15.80	0.31 ± 0.05	2.55*± 0.14
4.5	0.87 ± 0.01	0.19 ± 0.02	118.25 ± 12.27	0.33 ± 0.03	2.35 ± 0.07
One-way ANOVA					
*F* (*t*-test)	1.4257^NS^	1.9320^NS^	1.9319^NS^	2.7161^NS^	5.6638*
*p*	0.2837	0.1783	0.1783	0.0913	0.0118

**Notes:**

NS Non-significant.

* Asterisks indicate significant differences at 5% of probability, relative to the control by Dunnett’s test.

Means ± SEM (*n* = 4).

### Root and leaf fluoride content

**Figure 5:**
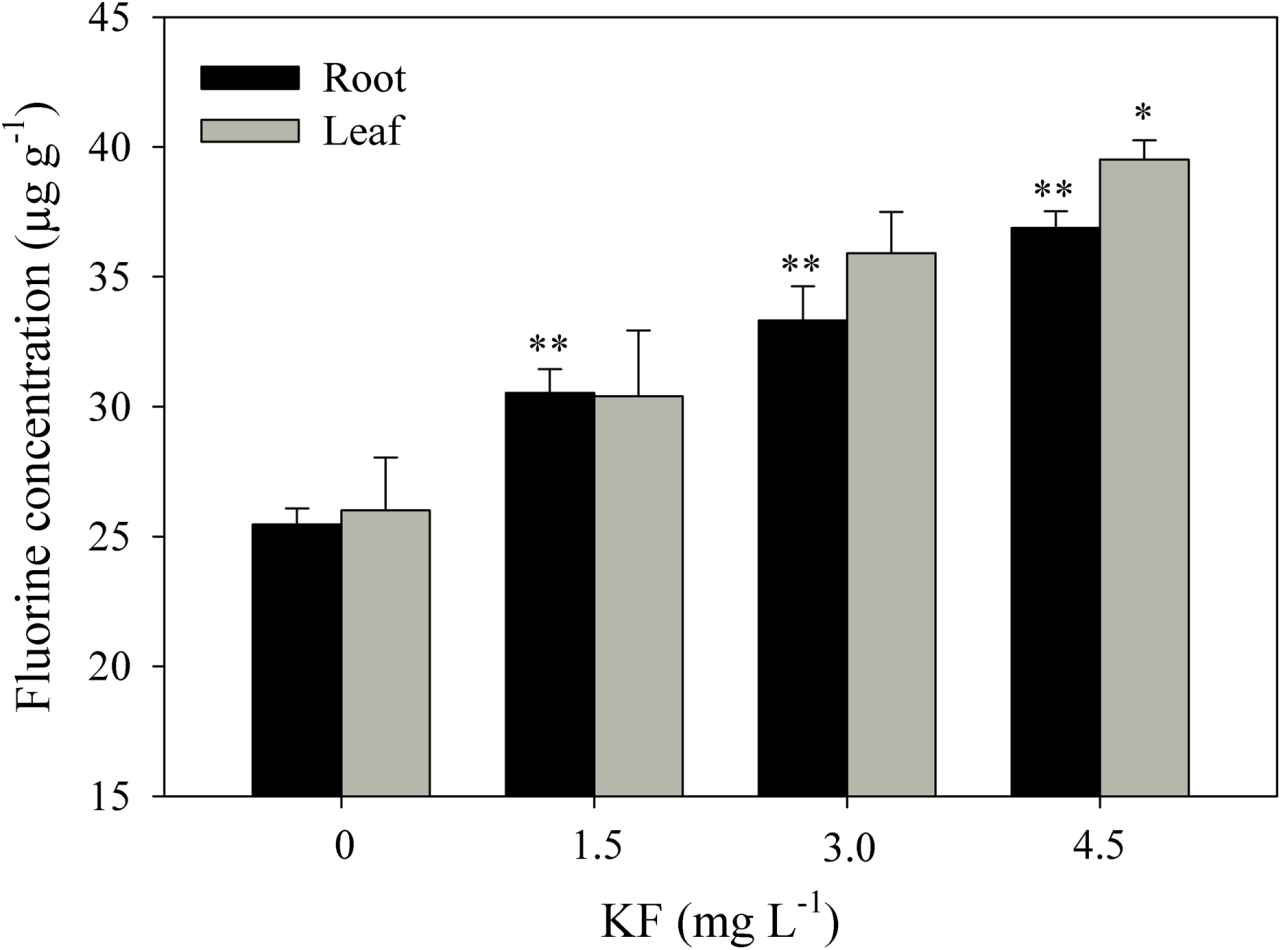
Fluoride content in *Ricinus communis* roots and leaves after 37 days of exposure to simulated rainfall containing KF. (0) Control, 1.5; 3.0 and 4.5 mg L^−1^ KF. Bars represent the mean ± SEM (*n* = 4). Asterisks indicate differences at 5% (*) and 1% (**) probability, relative to the control by Dunnett’s test. (NS), non-significant. One-way ANOVA Root (*F*-value 35.4011**; *p* < 0.0001) and Leaf (*F*-value 6.6645*; *p* = 0.0144).

Fluoride contents in roots were 24, 32 and 48% higher in the 1.5, 3.0 and 4.5 mg L^−1^ KF treatments when compared to the control (Fig. 5). For leaves, only 4.5 mg L^−1^ KF led to differences in relation to the control.

## Discussion

*Ricinus communis* morphoanatomical characteristics were not affected by the 1.5 mg L^−1^ F treatment. Similarly, Devi et al. (2016), who studied different plant species from regions near fertilizer factories presenting soil contaminated with up to 404 mg kg^-1^ of fluoride, concluded that F exposure did not alter the morphological characteristics of *Prosopis juliflora*, *Brachiaria distachya*, and *Scopharin dulci*. The authors also observed that these species displayed a high capacity to accumulate F in roots and leaves at concentrations ranging between 300 and 780 mg kg^-1^. Gao & Zhao (2014) reported that subcellular F distributions in tea plants play important detoxification roles. Most F can be sequestered in vacuole fractions, possibly reducing organelle toxicity and thereby preventing phytotoxicity to cellular structures. However, 3.0 and 4.5 mg L^−1^ fluoride led to visible injury symptoms with anatomical changes in *R. communis*. Direct F contact with leaves can cause visual symptoms such as chlorosis (yellowing) and leaf necrosis (Gupta et al., 2009). It is important to note that varying degrees of fluoride compound tolerance can be observed in plants, depending on the concentration and exposure time (Rey-Asensio & Carballeia, 2007). The application of 3 and 4.5 mg L^−1^ of KF caused evident formation of collapsed cells, mesophilic retraction with palisade parenchyma, spongy and mesophilic thickness reduction in *R. communis*. These results are likely due to changes in the turgor of mesophilic cells, giving them a flat and collapsed appearance (Sant’Anna-Santos et al., 2012; Sharma & Kaur, 2018). These data suggest that the association between tolerance potential and anatomical and physiological evaluations may be used as KF bioindicators.

Gas exchange remained constant in *R. communis*. In the natural environment, CO_2_ concentrations remain relatively unchanged. Stomata are sensitive to changes in CO_2_, responding to the mole fraction of CO_2_ in intercellular mesophyll spaces (Oguntimehin, Kondo & Sakugawa, 2010). In this case, stomata opening and closing is highly sensitive to atmospheric pollutant exposure and represents a protective mechanism to limit pollutant entry into leaves, despite the fact that this may result in lower photosynthetic rates (Rao et al., 1983; Cai et al., 2016). F triggers stomata closing in several species; nevertheless, *A*, *E*, *A/*C. and Φ_CO2_ remained constant in *R. communis*, suggesting that potassium fluoride did not trigger damage to Rubisco, the main enzyme involved in carbon fixation (Manter & Kerrigan, 2004; Walker, South & Ort, 2016). The maintenance (or increase) of photosynthetic efficiency under stressful conditions can be interpreted in terms of compensation and acclimatization, occurring while plants recover from damages caused by atmospheric pollutants (Bussotti, Strasser & Schaub, 2007; Duan et al., 2019). Nevertheless, prolonged stress and exposure to high KF may inhibit these defense mechanisms, compromising plant development.

Chlorophyll *a* fluorescence traits provide insights into photochemical PSII efficiency and the ability to tolerate environmental stresses (Baker et al., 2001). Fluoride causes physiological levels that affect chlorophyl *a* fluorescence in sensitive plants (Ghassemi-Golezani & Farhangi-Abriz, 2019). Nevertheless, little is known about the physiological responses of tolerant plants. It is expected that accumulated atmospheric pollutants would trigger abiotic stress responses in sensitive plants (Gorbe & Calatayud, 2012), which was not observed in *R. communis*. The absence of changes in Φ_PSII_, *F*_v_/*F*_m_, qP and ETR suggest maintenance of the physiological metabolism (Baker, Harbinson & Kramer, 2007). In particular, the increased qN indicates thermal dissipation of excess energy as a defense mechanism (Tomar & Jajoo, 2015) and to avoid photooxidative damage. Increases in non-photochemical quenching have also been detected in *Secale cereale* exposed to aluminum for short periods (Silva et al., 2012). KF exposure in tolerant species may result in qN recovery values close to those of control, and stabilized *F*_0_ suggests a tendency to maintain the balance between light level energy absorption and light energy use.

In addition, KF in *R. communis* led to the accumulation of starch grains in root cells and decreases in leaves. In toxic doses, F gives rise to higher amounts of starch in leaves, an effect related to the inhibitory effect of F on carbohydrate translocation from leaves to roots, leading to starch accumulation in chloroplasts (Sant’Anna-Santos et al., 2006). It is possible that KF treatments did not affect *R. communis* carbohydrate translocation, suggesting a greater tolerance of this species to KF. *R. communis* roots proved to be an important reserve organ in this species, directly related to the plant source/sink ratio when storing the starch produced in leaves. As observed in plants exposed to KF, starch accumulation in roots has been previously reported in heavy metals-treated plants (Eleftheriou et al., 2015). The reduced carbon reserve, by starch, in plants exposed to stress allows it to be used in the release of energy, sugars and metabolites which, in turn, may be used to protect the plants against oxidative stress (Thalmann & Santelia, 2017). Because of fluoride toxicity, changes at the physiological level affect chlorophyl *a* fluorescence in sensitive plants (Boukhris et al., 2015). Fluoride was absorbed by the root system at a rate proportional to increasing KF doses. F adsorbed by roots may become attached to cell wall components such as calcium or ionizable compounds. Previous studies have demonstrated that several species from semiarid regions accumulate F in both cytosolic and cell wall fractions (Baunthiyal & Sharma, 2012). This mechanism is crucial to improve fluoride tolerance in *R. communis* plants exposed to 1.5 and 3.0 mg L^−1^ KF. However, F can deconstruct root cells, promoting vein retraction and cellular collapse, as observed in *R. communis* after exposure to 4.5 mg L^−1^ KF. These changes indicate cell toxicity from the highest F accumulation in leaves and roots in the 4.5 mg L^−1^ treatment. The fluoride content in leaves and roots in the control treatment is derived from the substrate (25 mg kg^−1^). Furthermore, normal F levels in plant leaves usually range from 2 to 20 µg/g fluoride (Sant’Anna-Santos et al., 2014). It is important to emphasize that this subject deserves further investigation, due to the considerable amounts of fluoride-contaminated food ingested by humans (Zohoori & Maguire, 2016). These include grains, vegetables and byproducts of these raw materials grown in industrial areas presenting high F levels or irrigated with waters containing high F concentrations (Jha, Nayak & Sharma, 2011).

## Conclusions

*Ricinus communis* is potentially tolerant to potassium fluoride at 1.5 and 3.0 mg L^−1^, accumulating F in roots and leaves. The preservation of noninvasive variables, including visual effects, Φ_PSII_, Φ_CO2_, F_*v*_/F_*m*_ ETR, *A* and *A*/Ci are important tools that can be used as biomarkers of fluoride action for this species. Nevertheless, it should be emphasized that visual and anatomical alterations were observed at 4.5 mg L^−1^ exposure, suggesting that F may serve as a pollutant detector. *R. communis* displays potential as a tolerance bioindicator in F-contaminated environments, and can be used in environmental quality monitoring programs.

## Supplemental Information

10.7717/peerj.9445/supp-1 Supplemental Information 1 Fluoride concentration.

10.7717/peerj.9445/supp-2 Supplemental Information 2 Gas exchanges and chlorophyl a fluorescence.

10.7717/peerj.9445/supp-3 Supplemental Information 3 Morphoanatomical measurements.

10.7717/peerj.9445/supp-4 Supplemental Information 4 Starch accumulation.

## Additional Information and Declarations

### Data Availability

The following information was supplied regarding data availability: Raw data is available in the Supplemental Files.
